# Stabilization of Polyoxometalate Charge Carriers via Redox‐Driven Nanoconfinement in Single‐Walled Carbon Nanotubes

**DOI:** 10.1002/anie.202115619

**Published:** 2022-01-03

**Authors:** Jack W. Jordan, Jamie M. Cameron, Grace A. Lowe, Graham A. Rance, Kayleigh L. Y. Fung, Lee R. Johnson, Darren A. Walsh, Andrei N. Khlobystov, Graham N. Newton

**Affiliations:** ^1^ Nottingham Applied Materials and Interfaces (NAMI) Group GSK Carbon Neutral Laboratories for Sustainable Chemistry University of Nottingham Nottingham NG7 2TU UK; ^2^ Nanoscale and Microscale Research Centre University of Nottingham Nottingham NG7 2RD UK; ^3^ School of Chemistry University of Nottingham Nottingham NG7 2RD UK

**Keywords:** Carbon Nanotubes, Electrochemistry, Nanoconfinement, Polyoxometalates, Redox Materials

## Abstract

We describe the preparation of hybrid redox materials based on polyoxomolybdates encapsulated within single‐walled carbon nanotubes (SWNTs). Polyoxomolybdates readily oxidize SWNTs under ambient conditions in solution, and here we study their charge‐transfer interactions with SWNTs to provide detailed mechanistic insights into the redox‐driven encapsulation of these and similar nanoclusters. We are able to correlate the relative redox potentials of the encapsulated clusters with the level of SWNT oxidation in the resultant hybrid materials and use this to show that precise redox tuning is a necessary requirement for successful encapsulation. The host–guest redox materials described here exhibit exceptional electrochemical stability, retaining up to 86 % of their charge capacity over 1000 oxidation/reduction cycles, despite the typical lability and solution‐phase electrochemical instability of the polyoxomolybdates we have explored. Our findings illustrate the broad applicability of the redox‐driven encapsulation approach to the design and fabrication of tunable, highly conductive, ultra‐stable nanoconfined energy materials.

## Graphical abstract

The wide applicability of the redox‐driven encapsulation of polyoxometalates (POMs) within single‐walled carbon nanotubes is shown, demonstrating that the level of carbon oxidation and the efficacy of the filling are determined by the frontier orbital energies of the POM. Nanoencapsulation is found to confer significant stability to polyoxomolybdates despite their inherent lability.

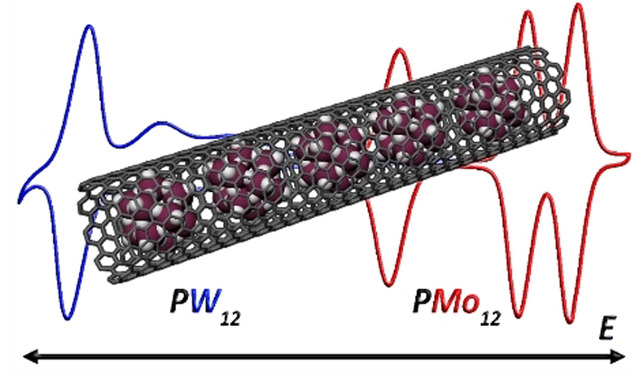

## Introduction

The exceptional electronic properties of carbon nanotubes (CNTs),^[1]^ coupled with their low density, high surface area and extraordinary mechanical properties,[^2^, ^3^] has inspired significant interest in their widespread use across a range of electronics and energy‐storage technologies. Applications range from logic components[^4^, ^5^, ^6^] and bioelectronic devices,[^7^, ^8^] to new electrode materials in lithium‐ion batteries and supercapacitors.[^9^, ^10^, ^11^, ^12^] The electronic properties of CNTs depend on their structure^[13]^ and can be modulated by chemical functionalization,[^14^, ^15^] electronic bleaching,[^16^, ^17^] or encapsulation of specific guest species.[^18^, ^19^, ^20^, ^21^] However, unlike fullerenes (their molecular analogues), CNTs do not undergo discrete redox processes owing to their extended atomic lattice and absence of discrete energy states. As such, CNTs typically exhibit capacitive behaviours similar to other conductive carbons.[^22^, ^23^] This precludes the precise tailoring of electronic properties required for some electrocatalytic or electronic applications,[^24^, ^25^] or access to additional faradaic (redox‐based) or pseudocapacitive charge storage processes that enable the development of high performance, energy‐storage materials.^[26]^ Conversely, the high aspect ratios and versatile surface chemistries of CNTs^[15]^ can facilitate their use as conducting substrates capable of “wiring” otherwise insulating redox‐active molecular species into new, tuneable, conductive nanocomposites.[^27^, ^28^]

Polyoxometalates (POMs) are a diverse class of multi‐redox active metal‐oxide nanoclusters typically based on tungsten, molybdenum or vanadium.^[29]^ Due to their enormously versatile structures and compositions,[^30^, ^31^, ^32^] which can be used to govern their functional properties, POMs have found widespread use as redox‐active components in high‐capacity electrode materials,[^33^, ^34^] high‐performance electrocatalysts,[^35^, ^36^] and molecular flash memory systems.^[37]^ Various methods have been used to immobilize POMs on CNT substrates, in order to improve their electrochemical addressability and stability in the solid state. Immobilization strategies include the electrostatic assembly^[38]^ and crystallization^[39]^ of POMs on the surface of CNTs to form layered coatings or adhered nanocrystals, the use of organic‐inorganic hybrid POMs to facilitate covalent binding of the POM directly to the nanotube surface,[^40^, ^41^] and non‐covalent adhesion mediated by π‐stacking “antenna” groups.[^42^, ^43^, ^44^, ^45^] Both approaches can be problematic; non‐covalent association of the POMs leads to relatively easy loss of the redox‐active clusters from the CNT surface, while covalent attachment leads to a loss of conductivity due to chemical modification of the sp^2^ carbon framework.^[39]^ The increased level of chemical complexity required for these systems can also result in a greater number of possible degradation pathways. Direct encapsulation of POMs within CNTs could offer an ideal solution, but this has typically proven challenging, due to the perceived mismatch between the anionic, hydrophilic POMs and the hydrophobic interiors of the CNTs. As such, there are very few examples describing the successful encapsulation of POMs within single‐walled carbon nanotubes (SWNTs), and these primarily focus on the use of stable, plenary polyoxotungstates.[^46^, ^47^, ^48^, ^49^] We recently reported that [PW_12_O_40_]^3−^ (**PW_12_**) and [P_2_W_18_O_62_]^6−^ (**P_2_W_18_**) spontaneously fill SWNTs under aqueous conditions, a process driven by an *in situ* redox reaction between the POMs and the SWNTs.^[50]^ The resultant hybrid materials (**PW_12_@SWNT** and **P_2_W_18_@SWNT** respectively, where @ indicates that the POMs are immobilized within the cavities of the SWNTs) exhibited reversible redox chemistry and were stable under harsh conditions and long‐term electrochemical cycling.

In this study, we employed the structurally analogous but electronically distinct polyoxomolybdate clusters, H_3_[PMo_12_O_40_] (**PMo_12_**) and K_6_[P_2_Mo_18_O_62_] (**P_2_Mo_18_**) to probe both the redox‐driven encapsulation mechanism and tuneable electrochemical properties and redox stability of these new hybrid materials. In comparison to the more commonly studied polyoxotungstate species, polyoxomolybdates typically exhibit significantly lower LUMO energies (i.e. more positive redox potentials) but are more structurally labile and suffer from considerably higher instability, particularly under redox cycling in aqueous conditions.[^51^, ^52^, ^53^] In doing so, we aimed to quantitatively confirm our charge‐transfer encapsulation hypothesis—where spontaneous oxidation of the SWNT host by the POM guest is a prerequisite for successful encapsulation—while also demonstrating the versatility of this approach with a view towards establishing clear design rules for the preparation of redox‐tuneable hybrid SWNT nanomaterials.

## Results and Discussion

The polyoxomolybdate clusters **PMo_12_** and **P_2_Mo_18_** were encapsulated in arc‐discharge prepared SWNTs (average diameter 1.4 nm) following our previously reported strategy.^[50]^ In a typical experiment, 20 mg of the commercially obtained SWNTs were first thermally annealed (600 °C for 30 mins in air) to give ca. 10 mg of opened SWNTs, which were then added directly to an aqueous solution (3 mL, 10 mM) of the corresponding POM, resulting in an immediate color change from yellow to green at the interface between the solution and the SWNT powder. The color change indicates that spontaneous charge transfer between the SWNT and the polyoxomolybdate had occurred, forming reduced POMs in solution and cationic SWNTs, and driving the formation of the POM@SWNT host–guest material by electrostatic interactions. The resulting mixture was then briefly sonicated and left to stir for 2 days, before it was filtered and washed with water to yield either **PMo_12_@SWNT** or **P_2_Mo_18_@SWNT** as a black solid.

**Figure 1:**
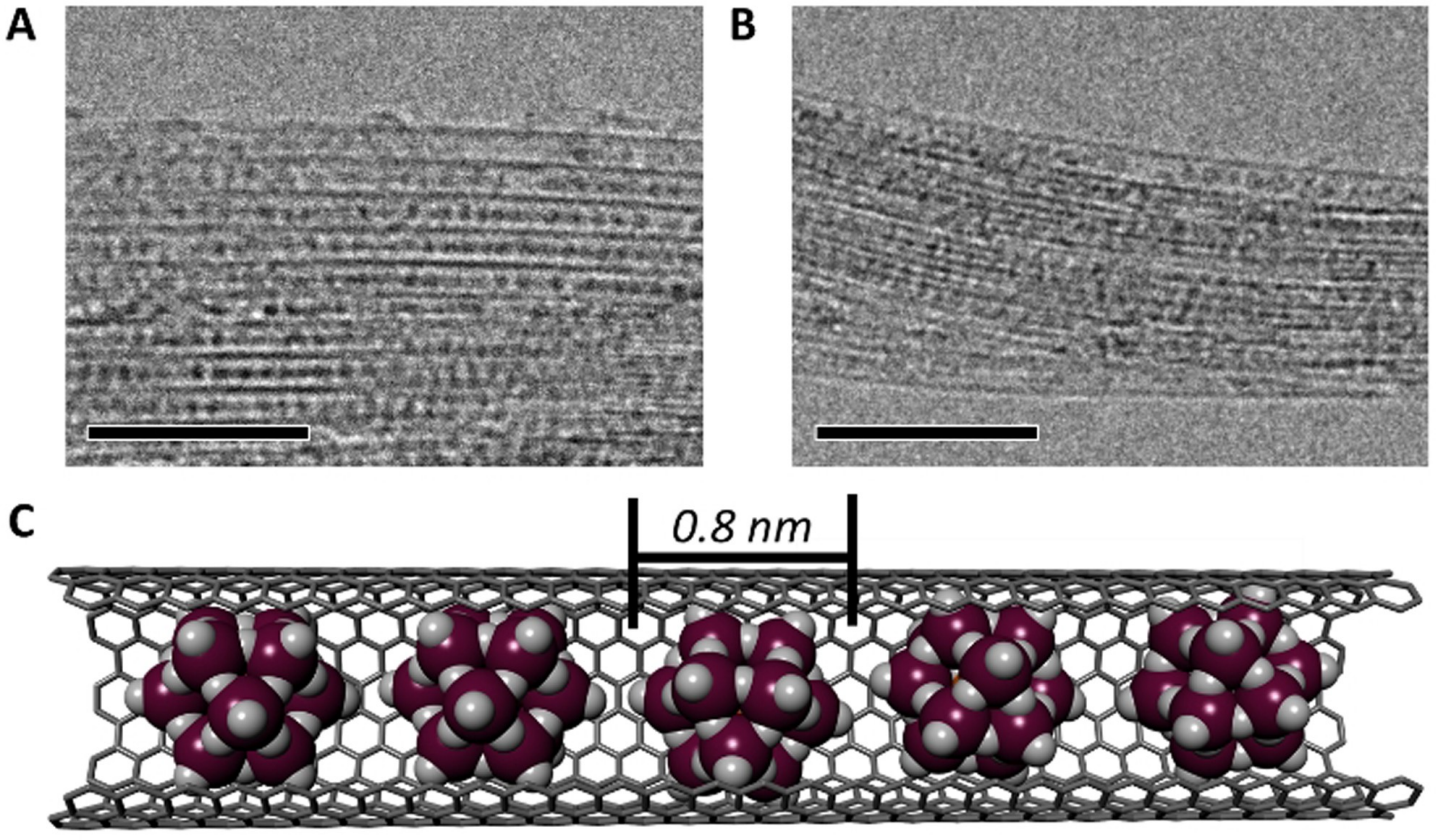
TEM images of **PMo_12_@SWNT** (A) and **P_2_Mo_18_@SWNT** (B). Images acquired with an accelerating voltage of 80 kV, scale bars 10 nm. C) Model of the **PMo_12_@SWNT** host–guest structure, molybdenum atoms in plum and oxygen in white.

**Figure 2:**
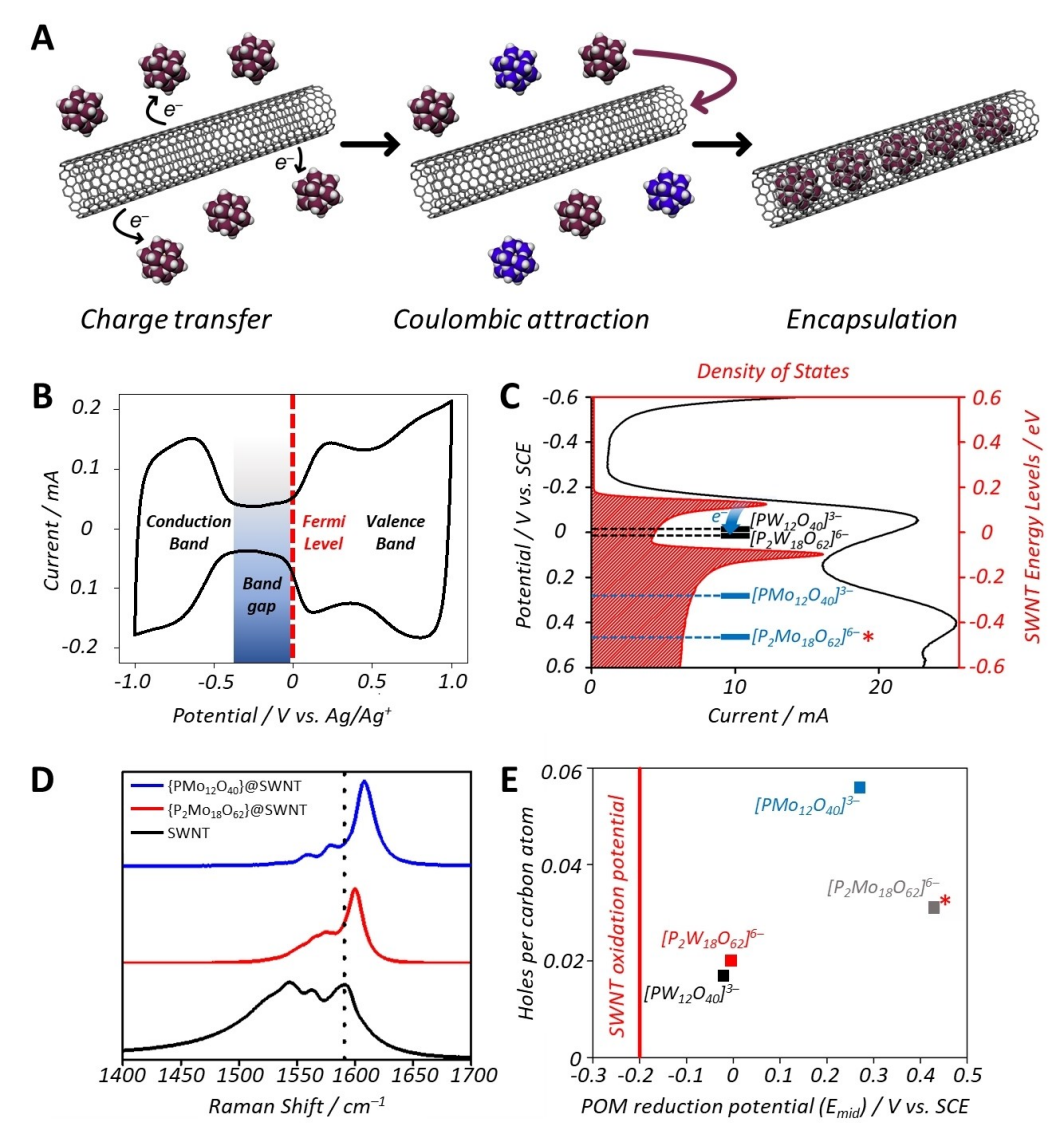
A) Scheme depicting the redox driven encapsulation of **PMo_12_** within SWNTs (plum colored POMs represent native, unreduced species and blue colored POMs represent reduced POMs with Mo^V^ centers which remain in solution). B) CV of **SWNT** thin film on glassy carbon electrode, with a dumbbell shape. Key features of the CV are labelled. C) Graph of the density of states of the SWNTs as a function of the applied potential relative to the reduction potentials of the POMs. D) Raman spectra (excitation 660 nm) of the **POM@SWNT** materials showing clear shifts in the position of the G‐band, indicative of the oxidation of SWNTs. Spectra were normalized to the intensity of the G band. E) The level of SWNT oxidation (holes per carbon atom) versus the reduction potentials of the encapsulated POMs. Note that the data obtained for **P_2_Mo_18_@SWNT** includes a contribution from POMs adsorbed on the external surface of SWNTs and is therefore not directly comparable to the other samples.

High‐magnification transmission electron microscopy (TEM) of **PMo_12_@SWNT** confirmed the encapsulation of **PMo_12_** (Figure 1A) and indicated that virtually all SWNTs had high POM loadings. Encapsulated POM clusters were clearly visible due to their high contrast (*Z*_Mo_=42) relative to the nanotube sidewalls (*Z*_C_=6), where discrete, closely packed, uniform species approximately 0.8 nm in diameter were observed (Figure 1C), in good agreement with the crystallographic diameter of **PMo_12_** (ca. 1 nm, though note that terminal oxygen are not typically observed with TEM). Similar to their polyoxotungstate analogues,^[54]^ the discrete species appeared to rapidly condense to larger nanoparticles during TEM observations, indicating that O atoms had dissociated from the POMs under the electron beam (Figure S1). Raman spectroscopy of **PMo_12_@SWNT** also provided strong evidence for POM encapsulation. The intensity of the band associated with the radial breathing mode (RBM) of the SWNT was strongly suppressed relative to that of the graphitic (G) band, (Figure S2) which itself had shifted significantly upon encapsulation and changed shape (Figure 2D, see below). Energy dispersive X‐ray (EDX) analysis of **PMo_12_@SWNT** bundles gave a P:Mo:O ratio of 1.1:12.0:40.3 (Figure S3), which is in excellent agreement with the expected values (1:12:40). Furthermore, X‐ray photoelectron spectroscopy (XPS) yielded atomic percentages in broad agreement with those obtained by EDX analysis and Mo 3d binding energies of 233.2 and 236.3 eV (Table S1, Figure S4), which are slightly positively shifted relative to that of the free POM and correspond well to a Mo oxidation state of +6.^[55]^ These observations indicate that the **PMo_12_** was chemically unchanged by encapsulation within the SWNTs and suggests that the POM was encapsulated exclusively in its native oxidation state (i.e. [PMo^VI^_12_O_40_]^3−^). This can be explained by considering the specifics of the electron‐transfer driven encapsulation mechanism and is discussed in more detail below.

TEM of **P_2_Mo_18_@SWNT** also confirmed successful encapsulation of the **P_2_Mo_18_** POMs within the SWNTs (Figure 1B). This was further corroborated by Raman spectroscopy, which showed similar relative suppression of the RBM bands to that seen for the **PMo_12_@SWNT** sample (Figure S2), coupled with a blue shift in the G band. TEM analysis of **P_2_Mo_18_@SWNT** also revealed that crystallites with dimensions ranging from a few hundred nm to approximately 10 μm had formed on the exterior of the SWNTs (Figure S5). These features were unique to **P_2_Mo_18_@SWNT**, were not observed in any of the hybrid materials formed using **PMo_12_** or tungsten analogues,^[50]^ and could not be removed easily, even after sonication in NaOH (which typically decomposes such clusters). Although it remains unclear as to why only **P_2_Mo_18_** both filled and decorated the exteriors of the SWNTs, we do note that this behavior is similar to that reported by Streb, Song and co‐workers, who described the ultrasonication‐driven formation of periodically‐arranged POM nanocrystals on similar SWNTs.[^39^, ^56^] As such, although there is clear evidence for the successful encapsulation of **P_2_Mo_18_** in the SWNTs, the challenge in interpreting our analysis of **P_2_Mo_18_@SWNT** in order to successfully deconvolute the effects of both encapsulated and surface‐bound POM means that we will focus the remainder of our discussion on **PMo_12_@SWNT**. Additional characterization of **P_2_Mo_18_@SWNT** can be found in the Supporting Information.

Cyclic voltammetry of both “empty” SWNTs and the discrete POM clusters was used to study the relative positions of the conduction and valence bands of the SWNTs and the LUMOs of the POMs. A cyclic voltammogram (CV) of empty SWNTs deposited on a glassy carbon (GC) electrode showed the characteristic “dumb‐bell” shape expected for these nanomaterials (Figure 2B),^[23]^ from which the position of the conduction and valence bands can be identified. Solution‐state CVs of each POM are typical of previous literature reports,^[51]^ and find that the mid‐point potentials (*E*_mid_) of the first reductions of the POMs ranged from *−*0.02 V vs. SCE for **PW_12_** to ca. +0.3 V vs. SCE for **PMo_12_** (see footnote,^[57]^ and Figure 2C, Figure S6 and Table S2), all of which are more positive than the top of the valence band of the SWNT (Figure 2C). This permits spontaneous charge transfer from the SWNTs to the relatively lower lying LUMOs of the POMs, oxidizing the SWNT and facilitating the electrostatically‐driven encapsulation of the POM clusters (Figure 2A), counteracting the otherwise highly unfavorable interaction between the hydrophobic SWNT interior and the hydrophilic POM.[^23^, ^58^]

As a useful control and proof of principle, we exchanged the K^+^ cations in **P_2_W_18_** for tetra‐*n*‐butylammonium (TBA^+^) ions and measured the solution‐state voltammetry of the resulting (TBA)_6_[P_2_W_18_O_62_] (**TBA‐P_2_W_18_**) cluster in acetonitrile (note that the TBA^+^ salt is not water soluble). The voltammetric analysis indicated that the first reduction potential of the POM was over 400 mV more negative than the edge of the valence band of the SWNTs when measured under the same conditions (Figure S7), with the change in reduction potential likely due to both cation effects^[59]^ and a change in the solvent dielectric (acetonitrile). The result of this meant that that spontaneous charge transfer between the nanotube and the POMs was no longer favored. When freshly annealed SWNTs were then added to an acetonitrile solution of **TBA‐P_2_W_18_**, no color change was observed, nor was any POM encapsulation identified using TEM, EDX or Raman spectroscopy analysis (Figure S8–10). This observation thus offers clear “design‐rules” for the preparation of similar hybrid nanomaterials in the future, based on careful matching of the redox potentials of both the CNT host and molecular guests.

Raman spectroscopy can also be used to probe the electronic state of the oxidized SWNTs after reaction with the POMs, as blue‐shifts in the G‐band can be correlated directly with the number of holes formed per carbon atom.[^60^, ^61^] In all cases, the Raman spectra of the POM@SWNT hybrid materials showed positive shifts in the G‐band relative to that of the empty SWNTs (Figure 2D) which was also accompanied with both a change in the peak shape and a relative suppression of the RBM mode, suggesting a change in the resonance conditions of the SWNTs due to their oxidation. (Figure S2). The Raman spectrum of **PMo_12_@SWNT** showed a positive G‐band shift of 18 cm^−1^ relative to that of the native SWNT, which corresponds to the overall oxidation of the nanotube by 0.056 holes/C atom. This is considerably higher than the shifts previously observed for the polyoxotungstate‐based hybrids (5.5 cm^−1^ or 0.017 holes/C for **PW_12_@SWNT**, and 6.5 cm^−1^ or 0.02 holes/C for **P_2_W_18_@SWNT**),^[50]^ and correlates well with the increased oxidative driving force of the SWNTs by reduction of the polyoxomolybdates in solution (≈500 mV) than of the polyoxotungstates (≈200 mV) (Figure 2E).

Based on the XPS data presented above (which is in full agreement with our previous study),^[50]^ the encapsulated POMs, which act as a charge‐balancing anions within the oxidized SWNTs, are found exclusively in their native d^0^/Mo^6+^ oxidation states. Upon encapsulation, the POMs shed their cations and become desolvated, an effect that is most clearly demonstrated for **P_2_W_18_@SWNT**, in which no K^+^ was detected by EDX analysis.^[50]^ This is to be expected, given the similarity between the diameters of the SWNT and **P_2_W_18_**, making encapsulation of K^+^ cations alongside the POMs sterically challenging (note that this is also likely true of **P_2_Mo_18_@SWNT**, but due to the crystallization of additional POM on the nanotube exterior K^+^ can be detected in the bulk material by both EDX and XPS analysis—see Supporting Information). The fact that the encapsulated POMs seem to be exclusively in their d^0^ state may be explained by considering both entropic and charge balance factors. In the former, the higher degree of solvation of the the d^0^ POMs,[^59^, ^62^] makes the encapsulation of these species, rather than the reduced anions (which pair more strongly with associated cations to the exclusion of solvent), more entropically favorable. In the latter, given that strong experimental evidence exists to show that additional cations are not encapsulated within the SWNT alongside the POM (i.e., the positively charged nanotube provides charge balance for all encapsulated anions), the only available reservoir for the additional cations liberated by encapsulated POMs are the reduced clusters in solution, themselves formed during the spontaneous oxidation of the nanotube. A concerted process whereby d^0^ POMs “hand‐over” their associated cations to reduced POMs in the vicinity of the SWNT as they are encapsulated is therefore the most plausible method to retain overall charge‐balance across the system as a whole.

**Figure 3:**
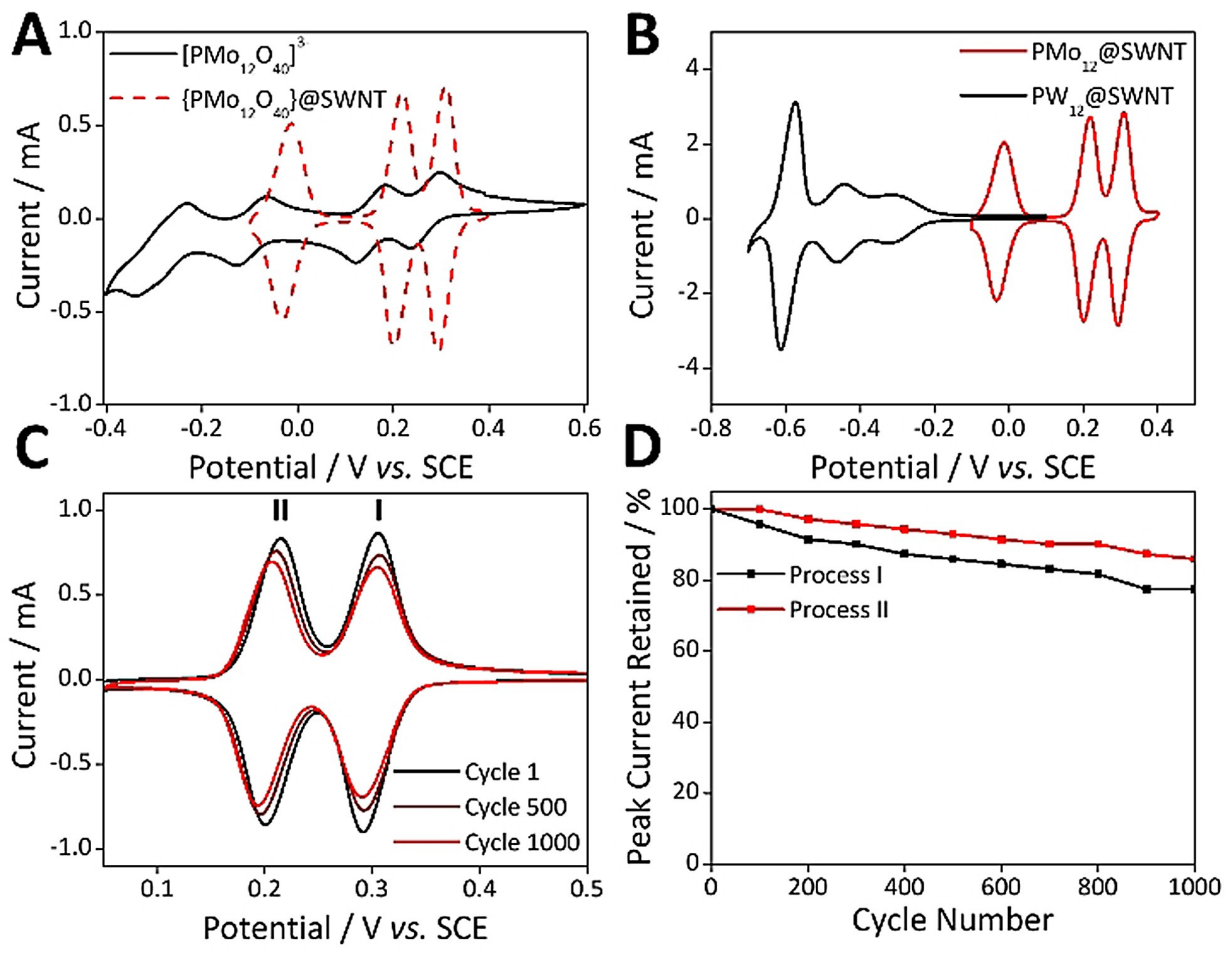
A) CV of **PMo_12_** (black trace) vs. **PMo_12_@SWNT** (dashed red trace) (each measured in their stable potential window). B) CV comparison of **PMo_12_@SWNT** (red) vs. **PW_12_@SWNT** (black) demonstrating the range of redox chemistries accessible in the **POM@SWNT** systems. C) The 1^st^ (black), 500^th^ (maroon), and 1000^th^ (red) voltammetric cycles of PMo_12_@SWNT. D) Plot illustrating the decay in peak currents (process I, black & process II, red) observed over 1000 charge–discharge cycles of **PMo_12_@SWNT**.

The redox chemistry of the **PMo_12_@SWNT** hybrid material was assessed by voltammetric analysis of films immobilized from aqueous dispersions (1 wt.% with 3 wt.% PTFE binder) in 1.0 M H_2_SO_4_ on a glassy carbon working electrode. The electrochemical cell contained a Pt counter electrode and a saturated calomel reference electrode. The electrochemical activity of the encapsulated **PMo_12_** cluster was fully retained between −0.1 and 0.6 V, and three reversible redox processes were observed with small positive shifts in their *E*_mid_ relative to those of solution‐phase **PMo_12_** (Figure 3A—note that the peak current intensities of free **PMo_12_** in solution and when encapsulated within SWNTs and deposited on the electrode surface cannot be directly compared, as described by the Randles–Ševćik equation).^[63]^ For **PMo_12_@SWNT**, scanning beyond negative potentials of ca. −0.3 V negatively impacted the observed electrochemical reversibility of the system, though this was subsequently recoverable by restraining the cycling window to between −0.1 and 0.5 V (Figure S11). The accessible processes were approximately 600–1000 mV more positive than the reductions of the analogous **PW_12_@SWNT** (Figure 3B). Analysis of the full width half maximum (FWHM) values for the observed redox process gave values between 42–57 mV. The theoretical value for a surface‐confined redox species is 90.6/*n* mV,^[63]^ where *n* is the number of electrons transferred during each process, meaning that each process involved the transfer of 2 electrons, matching the behavior of **PMo_12_** in solution. In addition, the peak‐to‐peak separation for each process, Δ*E*_p_, ranged from 15–20 mV at a scan rate of 100 mV s^−1^ and the peak reduction/oxidation currents increased linearly with increasing scan rate in the range 50–500 mV s^−1^ (Figure S12). These observations are also expected during voltammetric analysis of a surface‐confined redox‐active species,^[63]^ and demonstrate that the POMs were in intimate electrical contact with the GC electrode. Note that Δ*E*_p_ increased with increasing scan rate in the range 50–500 mV s^−1^, indicating that the processes were electrochemically quasi‐reversible (Figure S13). Using the charge passed during reduction of the adsorbed POMs, the surface concentration of the POMs was calculated to be approximately 11 nmol cm^−2^, in good agreement with our previously reported POM surface concentrations suggesting that increased oxidation of the SWNT does not result in an increased loading of the POM species. Repeated potential cycling of the composite material was carried out to test the electrochemical stability of **PMo_12_@SWNT**. 86 % of the initial peak currents was retained after cycling the material between 0.0 and 0.5 V in 1.0 M H_2_SO_4_ 1000 times (Figure 3C,D). This electrochemical stability far exceeded that of the tungsten‐based analogue **P_2_W_18_@SWNT**, which retained a maximum of 58 % of its initial peak current over 1000 cycles,^[50]^ and is even more noteworthy given that **PMo_12_** typically requires stabilization by addition of organic solvent to prevent hydrolysis or surface confinement to obtain stable voltammetric cycling.[^51^, ^52^, ^53^] Note that cycling the **PMo_12_** cluster in solution under similar conditions leads to adsorption of a small amount of POM onto the GC electrode surface, which confers some additional cycling stability, but nevertheless shows significantly poorer definition of redox waves and peak currents several orders of magnitude less than that of the encapsulated species. Additionally, the SWNT was able to confer considerable chemical stability to the encapsulated POM. Almost all POMs are hydrolytically unstable at high pH,^[29]^ decomposing rapidly to small and typically redox‐silent oxoanions (i.e. MoO_4_^2−^). Interestingly, while **PMo_12_@SWNT** is essentially redox‐silent when measured in 1.0 M NaOH electrolyte over 10 cycles (Figure S15), when the same **PMo_12_@SWNT**‐functionalized electrode was added to a cell containing fresh 1.0 M H_2_SO_4_ the POM‐based redox processes gradually reappeared over 100 cycles (Figure S16). This indicates that while the encapsulated POMs are electrochemically silent at high pH, they are also protected from hydrolysis under these conditions by encapsulation within the SWNTs. This result is similar to our previous work, where TEM analysis of **P_2_W_18_@SWNT** after exposure to base confirmed the POMs were still discrete species within the SWNTs.^[50]^

This confirms the excellent chemical stability of **PMo_12_@SWNT**, but also highlights an interesting disparity between the redox behavior of the Mo‐ and W‐based hybrid materials, suggesting that the proton coupled electron transfers (PCETs) exhibited by POMs played a particularly important role in the observed redox chemistry of the **PMo_12_@SWNT** material. This can be seen clearly by comparing the differences between the voltammograms of free and encapsulated **PMo_12_** and **PW_12_**. In the former, encapsulation of the POM within the SWNT had a marginal effect on the electrochemical properties of the cluster (Figure 3A), shifting all three reversible 2e^−^ redox processes to slightly more positive potentials, with shifts ranging from 31 to 71 mV. Conversely, the voltammograms of **PW_12_** and **PW_12_@SWNT** are dissimilar; encapsulation caused significant negative shifts in the potentials of the first two, non‐proton‐coupled 1e^−^ redox waves (of 282 and 197 mV, respectively), while the third 2e^−^ process remained at almost the same potential as that of the free cluster.^[50]^ This behavior maps precisely onto the known proton‐coupled electron transfer (PCET) redox chemistry of both POMs,^[51]^ where those processes which are proton‐coupled 2e^−^ transfers maintain their original redox potentials, and those which are not typically proton‐coupled are shifted negatively. This suggests that under the standard electrochemical conditions the nanotube cavity is strongly acidic, an observation that is further supported by the observation of 3 redox processes in the voltammogram of **P_2_W_18_@SWNT**.[^50^, ^51^] That non‐PCET mediated redox processes shifted negatively may be due to increased coulombic forces experienced by the encapsulated and desolvated POMs. The dynamics of cation transfer within these hybrid materials is of both fundamental interest, and highly significant for applications in energy storage, and further study into the properties of these materials is ongoing in our laboratories. Despite the increased chemical and electrochemical stability, the thermal stability, as measured by thermal gravimetric analysis (TGA), was found to decrease in the hybrid material, with the decomposition of the SWNT host occurring at approximately 100 °C lower than pristine SWNTs (Figure S17). This indicates that the encapsulated POMs may facilitate SWNT decomposition, a finding in line with a previous report of the thermal stability of a MoO_*x*_ nanoparticle/carbon nanotube hybrid material.^[64]^

## Conclusion

In summary, the mechanism of charge transfer‐driven encapsulation of the POM clusters has been verified by exploring the effects of encapsulation of POM clusters with significantly lower (**PMo_12_**, **P_2_Mo_18_**) and higher (**TBA‐P_2_W_18_**) LUMO energies than the top of the SWNT valence band, allowing the reduction potential of the POM to be correlated directly to its encapsulation via oxidation of the nanotube. Encapsulation of **PMo_12_** within SWNTs offered a significant enhancement in the cycling stability of the cluster. Establishing clear design rules for the preparation of these highly stable materials is hugely important, as it now allows the redox properties of the resulting hybrid materials to be tuned across a broad potential range. It is important to note that there is already a huge number of reported POM clusters, including those functionalized with additional heterometals or organic groups with dimensions and redox properties which render them theoretically suitable for incorporation into the same 1.4 nm SWNTs used here. Clearly, the suitability of a wide range of other redox molecules and nanotubes of differing sizes and chemistries can also be explored using the strategies described in this paper. As such, we hope that the insights reported here will serve as a useful guide for the straightforward and scalable development of new, highly stable redox‐active host–guest CNT nanocomposites.

## Conflict of interest

The authors declare no conflict of interest.

1

## Supporting information

As a service to our authors and readers, this journal provides supporting information supplied by the authors. Such materials are peer reviewed and may be re‐organized for online delivery, but are not copy‐edited or typeset. Technical support issues arising from supporting information (other than missing files) should be addressed to the authors.

Supporting Information

## Data Availability

The data that support the findings of this study are available in the supplementary material of this article.
