# Supplementary material for: Stabilization of Polyoxometalate Charge Carriers via Redox‐Driven Nanoconfinement in Single‐Walled Carbon Nanotubes
Source: Angew Chem Int Ed Engl. 2022 Jan 3;61(8):e202115619. doi: 10.1002/anie.202115619 (PMC9304274; doi:10.1002/anie.202115619)
Supplement: Supplementary file 1 — Supporting Information [file ANIE-61-0-s001.pdf]

Angewandte Chemie
Eine Zeitschrift der Gesellschaft Deutscher Chemiker

Supporting Information

Stabilization of Polyoxometalate Charge Carriers via Redox-Driven Nanoconfinement in Single-Walled Carbon Nanotubes

*J. W. Jordan, J. M. Cameron, G. A. Lowe, G. A. Rance, K. L. Y. Fung, L. R. Johnson, D. A. Walsh, A. N. Khlobystov, G. N. Newton**

Experimental

All common reagents and solvents were used as received from Sigma-Aldrich, Acros Organics or Thermo Fisher. Single walled carbon nanotubes (P2-SWNT) were purchased from Carbon Solutions Inc. $H_3[PMo_{12}O_{40}]$ (**PMo_{12}**) was synthesized following a previously reported method.[1] Briefly, MoO_3 (14 g, 0.097 moles) was dissolved in water (150 mL). H_3PO_4 (14.8 M, 570 µL) was added to the resultant green suspension. The mixture was refluxed for 3 hours at 110 °C, giving a bright yellow suspension. The suspension was filtered whilst still hot, giving a clear yellow solution. The solvent was reduced with heat and allowed to stand overnight yielding a yellow precipitate. This was filtered and washed with cold ethanol (5 mL) and cold water (5 mL) giving a yellow solid (5.37 g, 37%) ^{31}P NMR (D_2O) (δ, ppm) -3.88 (s). The synthesis of $K_6[P_2Mo_{18}O_{62}]$ (**P_2Mo_{18}**) was adapted from a previously reported method.[2] Briefly, $Na_2MoO_4.2H_2O$ (20 g, 0.083 moles) was dissolved in water (30 mL). HCl (12.1 M, 16.5 mL) was added to the solution upon which a white precipitate formed, which was dissolved with stirring, giving a yellow solution. H_3PO_4 (14.8 M, 3 mL) was then added to the solution. The resultant yellow solution was refluxed overnight at 110 °C, resulting in a deep orange solution. Once the reaction had cooled to room temperature KCl (27 g, 0.36 moles) was added, producing a yellow precipitate. This precipitate was filtered and washed with cold ethanol (5 mL) and cold water (5 mL) giving a yellow solid (3.50 g, 25%). ^{31}P NMR (D_2O) (δ, ppm) -3.14 (s).

The synthesis of **POM@SWNT** materials was carried out by dissolving the appropriate POM cluster (**PMo_{12}** or **P_2Mo_{18}**) in water (3 mL) to give a 10 mM solution. Commercially obtained SWNTs (20 mg) were heated to 600 °C in air for 30 minutes yielding a black solid (10 mg), which was cooled and then added to the POM solution. Upon the addition, the rapid formation of a green solution was observed at the solid-liquid interface. The suspension was then sonicated for approximately 5 minutes and stirred at room temperature for 48 hours. The resulting suspension was then filtered through a PTFE membrane (pore size 0.2 µm) to yield the product **POM@SWNT** as a black solid (typically 11-15 mg). For analysis methods, please see the Supporting Information.

Methods

Electrochemical Methods. Cyclic voltammetry and linear sweep voltammetry experiments were performed on an Autolab PGSTAT302N workstation using a typical three-electrode set up comprising a glassy carbon working electrode (28.3 mm^2) and platinum counter electrode. A saturated calomel electrode (SCE) was employed as the reference under aqueous conditions and a Ag/Ag^+ ($AgNO_3$) reference was employed in non-aqueous solvent conditions (referenced to Fc^+/Fc). Note that unless otherwise noted, for ease of reference all potentials quoted above are reported relative to that of the SCE. In a typical experiment, soluble analytes were dissolved in the supporting electrolyte to a concentration of 10 mM. Insoluble analytes were prepared as suspensions and then deposited *via* micropipette onto the working electrode surface. Typically, unfilled SWNTs were first sonicated for 15 minutes in a 10 mg/mL DMF suspension and **POM@SWNT** materials were likewise sonicated for 15 minutes in an aqueous 'ink' with 3 wt.% PTFE binder. After sonication, 8 µL of the resulting suspension was deposited onto the glassy carbon working electrode and allowed to air-dry.

Imaging Methods. TEM images were acquired on a JEOL 2100+ LaB_6 emission microscope and a JEOL 2100F field emission gun microscope with an accelerating voltage of 80 – 100 kV (stated as acquired). Samples were prepared by first dispersing them in isopropyl alcohol which were then drop-cast onto a copper grid mounted with a "lacey" carbon film. All TEM images were processed using Gatan Digital Micrograph, and quoted distances were measured by drawing a line profile and measuring the electron intensity histogram. EDX spectra were acquired during TEM imaging, using Oxford Instruments INCA X-Ray microanalysis systems.

Raman Spectroscopy. Micro-Raman spectroscopy was performed using a Horiba-Jobin-Yvon LabRAM HR spectrometer. Single-point spectra were recorded using a 100x objective, a 300-µm confocal pinhole, and a 660 nm (1.88 eV) laser at < 0.3 mW. The spectral resolution was 1.2 cm^{-1}. Instrument calibration was performed using the zero-order line and a standard Si(100) reference band at 520.7 cm^{-1}. Samples were prepared by depositing a small quantity of sample from a methanolic suspension onto Si(100) wafers. A typical spectrum was recorded by averaging 4-8 acquisitions, each of 5-30 s duration.

X-Ray Photoelectron Spectroscopy. XPS data was acquired with a Kratos AXIS ULTRA with a mono-chromated Al kα X-ray source (1486.6eV) operated at 10 mA emission current and 12 kV anode potential (120 W). Spectra were acquired with the Kratos VISION II software. A charge neutralizer filament was used to prevent surface charging. Hybrid –slot mode was used measuring a sample area of approximately 300 x 700 µm. The analysis chamber pressure was better than 5 x 10^{-9} mbar. Three areas per sample were analysed. A wide scan was performed at low resolution (Binding energy range 1400 eV to -5 eV, with pass energy 80 eV, step 0.5 eV, sweep time 20 minutes). High resolution spectra at pass energy 20 eV, step of 0.1 eV, and sweep times of 10 minutes each were also acquired for photoelectron peaks from the detected elements. The spectra were charge corrected to the C 1s peak set to 284.5 eV.

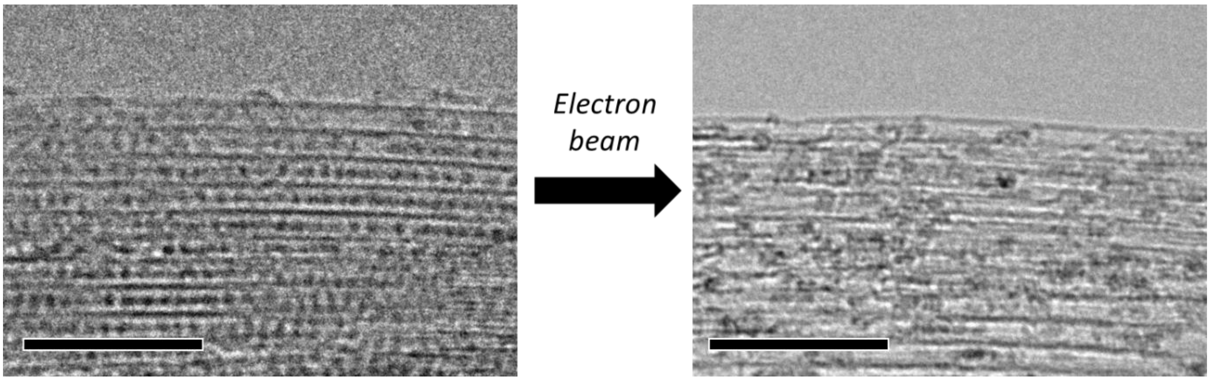

***Figure 1**. TEM images showing the effect of the incident electron beam on the structure of **PMo_{12}@SWNT**. Before irradiation uniform, discrete, molecular species (pristine **PMo_{12}**) can be observed. After extended irradiation non-uniform MoO_x species can be observed. Images acquired with an accelerating voltage of 80 kV, scale bars 10 nm.*

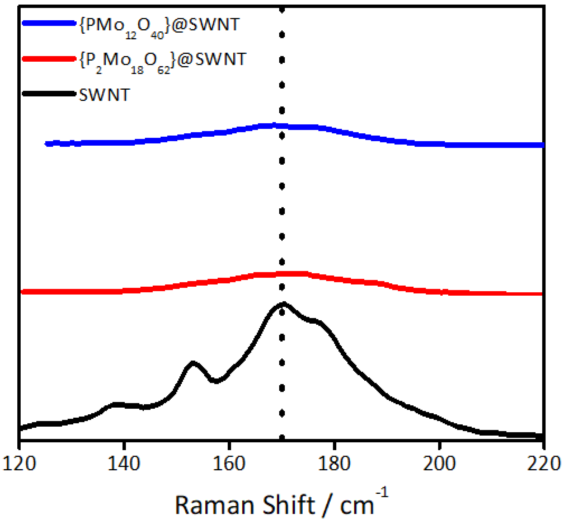

Figure S2. *Raman spectra highlighting the region associated with the 'radial breathing mode' (RBM) of empty* ***SWNTs****,* ***PMo_{12}@SWNT*** *and* ***P_2Mo_{18}@SWNT****. Data was acquired with an excitation energy of 660 nm.*

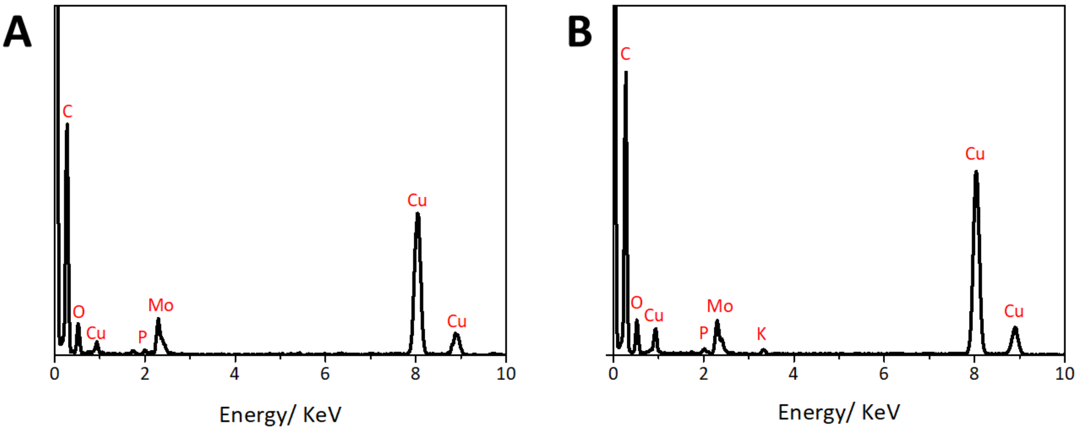

Figure S3. *EDX spectra of* ***PMo_{12}@SWNT*** *(A) and* ***P_2Mo_{18}@SWNT*** *(B) obtained from spot analysis of filled nanotubes during TEM analysis (note that copper is present due to the underlying TEM grid).*

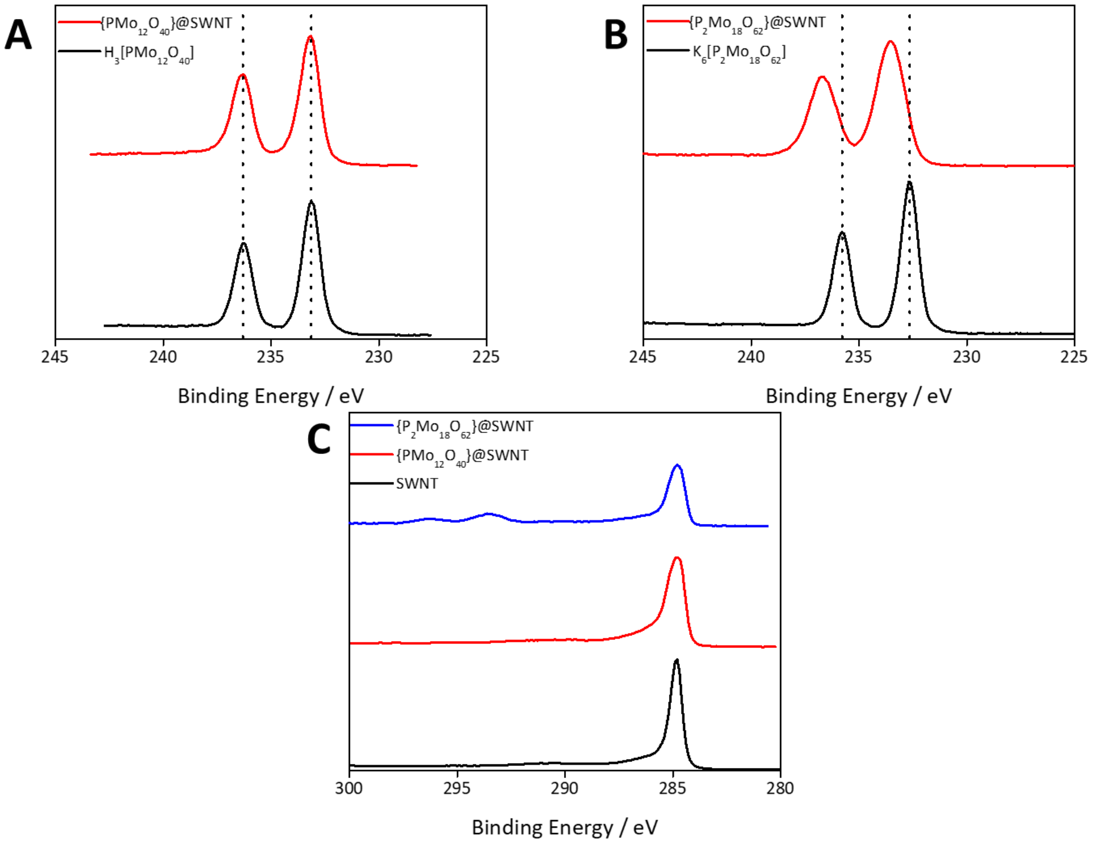

Figure S4. *(A) Mo 3d peaks of* ***$H_3[PMo_{12}O_{40}]$*** *and* ***PMo_{12}@SWNT****. (B) Mo 3d peaks of* ***$K_6[P_2Mo_{18}O_{62}]$*** *and* ***P_2Mo_{18}@SWNT****. (C) Carbon 1s peaks for empty* ***SWNTs, PMo_{12}@SWNT*** *and* ***P_2Mo_{18}@SWNT****.*

Table S1. *Elemental percentages calculated via XPS spectroscopic analysis*

SWNT				
Sample	C %	O %	Ni %	Y %
SWNT	93.5	4.8	1	0.5

Mo-POM							
Sample	C %	O %	Mo %	P %	K %	Ni %	Y %
$H_3[PMo_{12}O_{40}]$	41.8	41.5	15.6	1.2	-	-	-
$K_6[P_2Mo_{18}O_{62}]$	14.5	53	18.94	2.3	6.84	-	-

{Mo-POM}@SWNT							
Sample	C %	O %	Mo %	P %	K %	Ni %	Y %
$\{PMo_{12}O_{40}\}$@SWNT	85	12.3	2.6	0.2	-	-	-
$\{P_2Mo_{18}O_{62}\}$@SWNT	66.7	15.7	4.4	0.6	12.7	-	-

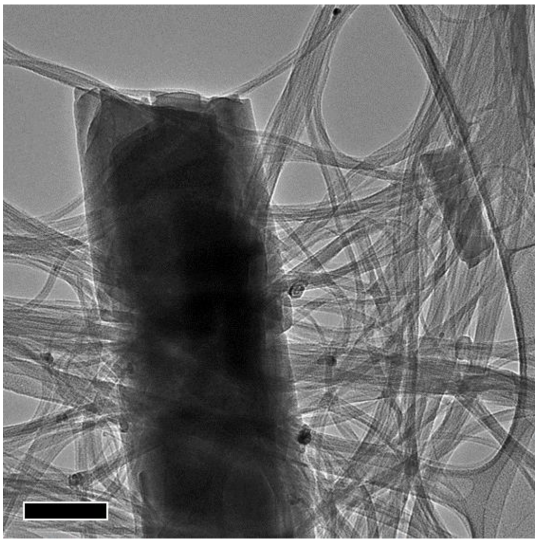

Figure S5. *TEM image of the* ***P_2Mo_{18}@SWNT*** *material showing a large, micron-sized crystal. Scale bar 100 nm. Image acquired with an accelerating voltage of 100 kV.*

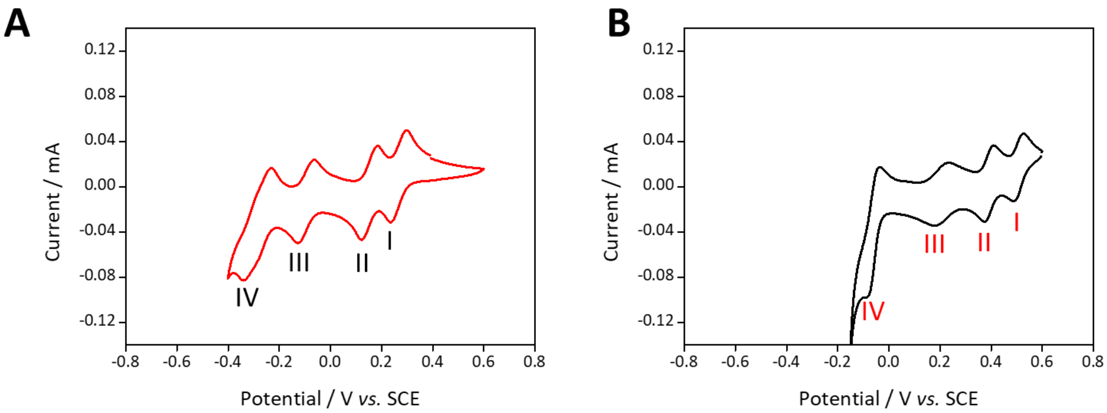

Figure S6. *(A) CV of* ***$H_3PMo_{12}O_{40}$*** *recorded in 0.1 M H_2SO_4 (aq.) with 80% MeCN (v/v). (B) CV of* ***$K_6[P_2Mo_{18}O_{62}]$*** *recorded in 1 M H_2SO_4. Both CVs acquired with a glassy carbon working electrode, saturated calomel reference electrode (SCE) and platinum counter electrode at a scan rate of 0.1 V s^{-1}.*

Table S2. *Electrochemical analysis of* ***PMo_{12}***

**$[PMo_{12}O_{40}]^{3-}$**		
**Peak**	**Assignment**	***$E_{1/2}$* / V**
I	$[PMo_{12}O_{40}]^{3-} + \mathbf{2e^-} + 2H^+ \rightleftharpoons [H_2PMo_{12}O_{40}]^{3-}$	0.271
II	$[H_2PMo_{12}O_{40}]^{3-} + \mathbf{2e^-} + 2H^+ \rightleftharpoons [H_4PMo_{12}O_{40}]^{3-}$	0.151
III	$[H_4PMo_{12}O_{40}]^{3-} + \mathbf{2e^-} + 2H^+ \rightleftharpoons [H_6PMo_{12}O_{40}]^{3-}$	-0.094
IV	$[H_6PMo_{12}O_{40}]^{3-} + \mathbf{4e^-} + 4H^+ \rightleftharpoons [H_{10}PMo_{12}O_{40}]^{3-}$	-0.282

Table S3. *Electrochemical analysis of* ***P_2Mo_{18}***

**$[P_2Mo_{18}O_{62}]^{6-}$**		
**Peak**	**Assignment**	***$E_{1/2}$* / V**
I	$[P_2Mo_{18}O_{62}]^{6-} + \mathbf{2e^-} + 2H^+ \rightleftharpoons [H_2P_2Mo_{18}O_{62}]^{6-}$	0.430
II	$[H_2P_2Mo_{18}O_{62}]^{6-} + \mathbf{2e^-} + 2H^+ \rightleftharpoons [H_4P_2Mo_{18}O_{62}]^{6-}$	0.330
III	$[H_4P_2Mo_{18}O_{62}]^{6-} + \mathbf{2e^-} + 2H^+ \rightleftharpoons [H_6P_2Mo_{18}O_{62}]^{6-}$	0.130
IV	$[H_6P_2Mo_{18}O_{62}]^{6-} + \mathbf{4e^-} + 4H^+ \rightleftharpoons [H_{10}P_2Mo_{18}O_{62}]^{6-}$	-0.125

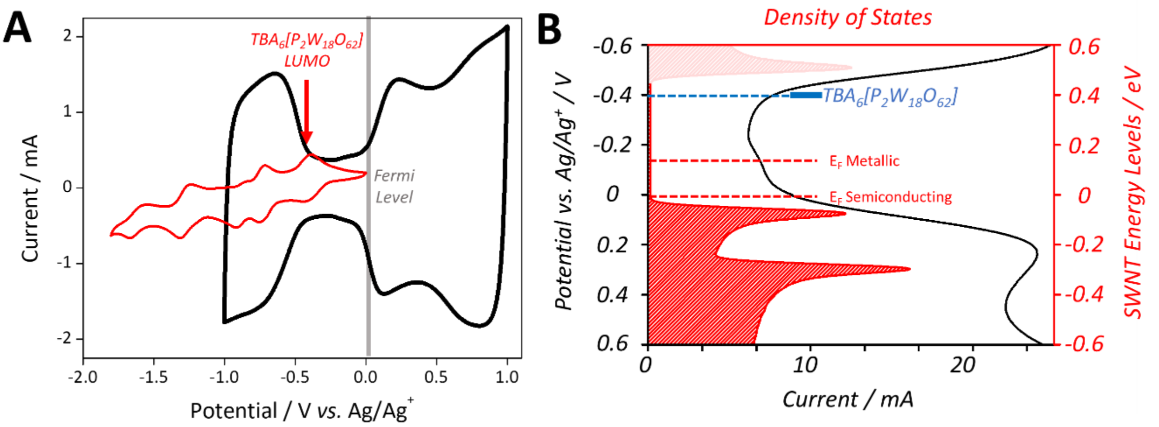

Figure S7. *(A) CVs of* ***$TBA_6[P_2W_{18}O_{62}]$*** *and empty* ***SWNTs*** *recorded in MeCN with a 0.1 M Bu_4NPF_6 supporting electrolyte, with a glassy carbon working electrode, Ag^+/Ag reference electrode and platinum counter electrode. (B) comparison of the redox potentials and corresponding energy levels of the* ***SWNT*** *with the first available LUMO of* ***$TBA_6[P_2W_{18}O_{62}]$****, indicating that the reduction potential of the POM is too negative to spontaneously oxidise the SWNTs in MeCN solution.*

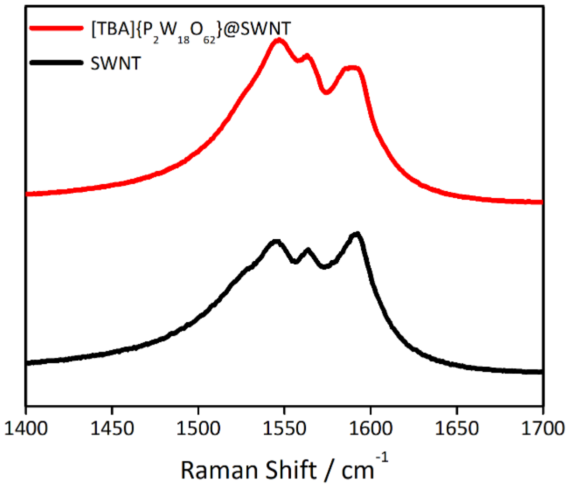

Figure S8. *Raman spectroscopic analysis highlighting the graphitic (G-)band of empty* ***SWNTs*** *and "****[TBA][$P_2W_{18}O_{62}$]@SWNT"****. In comparison with other samples (see Figure 2D, for example), the lack of a notable shift in the G-band energies indicates no significant interaction between the POM and the SWNT.Data gathered with an excitation energy of 660 nm.*

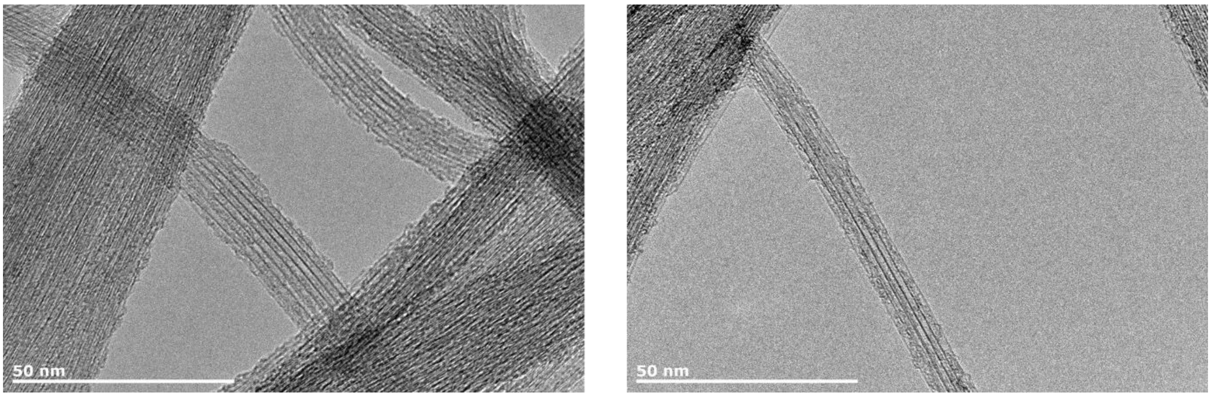

Figure S9. *TEM images of "****[TBA-$P_2W_{18}O_{62}$]@SWNT"*** *(i.e. the resultant solid material isolated from a mixture of $(TBA)_6[P_2W_{18}O_{62}]$ and SWNT in MeCN)*. In comparison to Figures 1A, B and S5, it is evident that there is no significant filling of the nanotubes, as expected based on the redox chemistry of both components.Images acquired with an accelerating voltage of 80 kV.

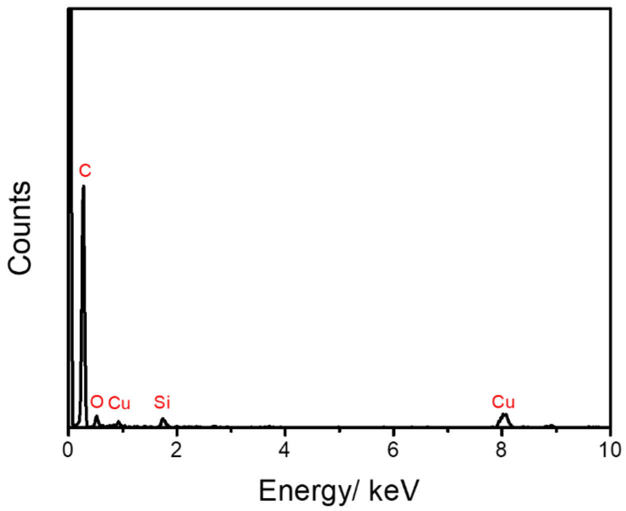

Figure S10. *EDX analysis of "**[TBA-P_2W_{18}]@SWNT"***, showing no significant contribution from W or P in the sample. Copper present due to the TEM grid.

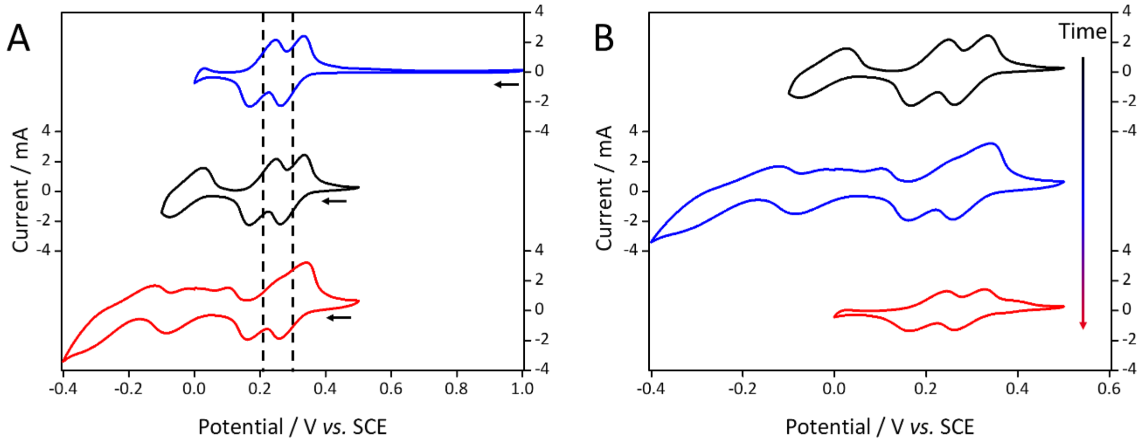

Figure S11. *(A) CVs of **PMo_{12}@SWNT** over different electrochemical windows. Arrows (inset) show the direction of the scan. (B) CVs over different electrochemical windows against time (indicated by the arrow inset) demonstrating the effect of narrowing the electrochemical window on the CV trace. CVs recorded with a GC working electrode, SCE reference electrode and platinum counter electrode at a scan rate of 0.1 V s^{-1}.*

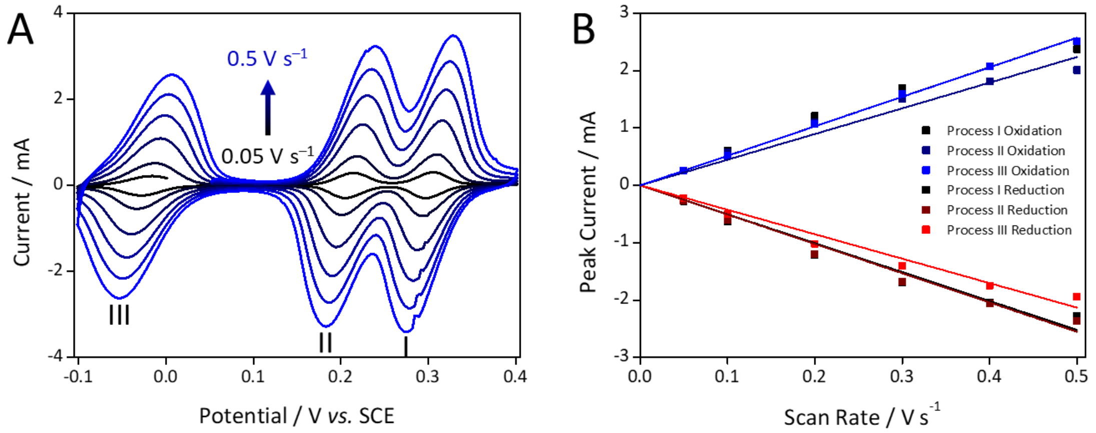

Figure S12. *(A) Cyclic voltammograms (CVs) of* ***PMo₁₂@SWNT*** *recorded at varying scan rates. (B) Peak current vs. scan rate for the processes shown in A, showing a linear relationship which is indicative of surface confined redox behaviour.*

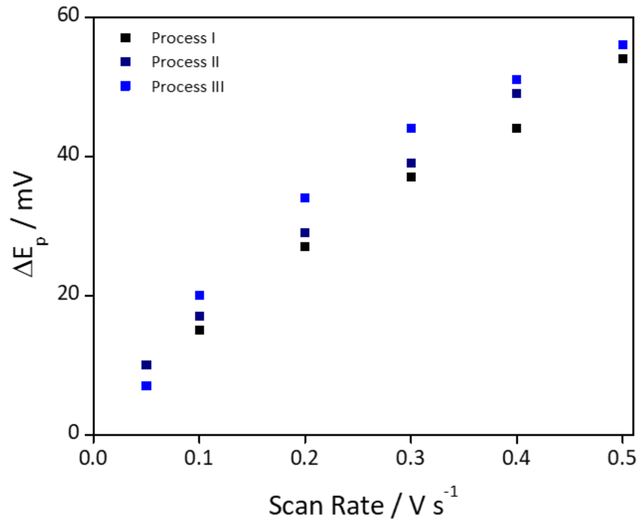

Figure S13. *Peak-to-peak separations (ΔE_p) vs. scan rate for* ***PMo₁₂@SWNT****, confirming the electrochemical quasi-reversibility of the nanotube-confined POM redox processes.*

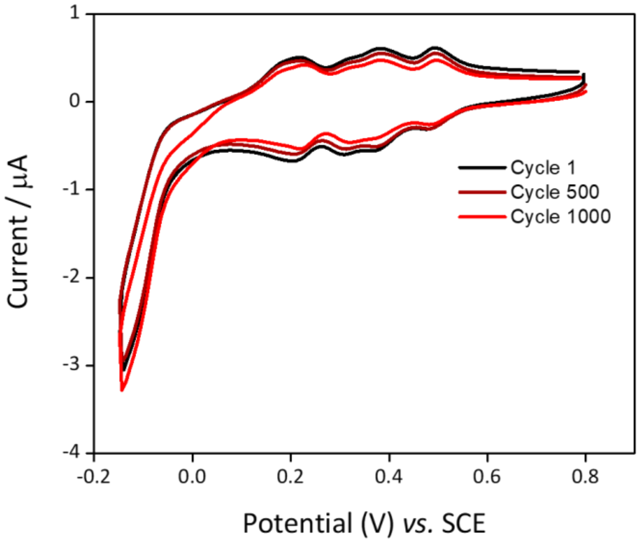

Figure S14. *CVs of **PMo_{12}** (25 mM) acquired in 1M H_2SO_4 taken over 1000 cycles. Note that this data suggests adsorption of a small amount of **PMo_{12}** onto the surface of the GC electrode (typical $\Delta Ep \approx 10$ mV) which offers some stabilisation under redox cycling, though peak definition and current is very poor compared to that of **PMo_{12}@SWNT** (Fig 3). CVs acquired with a GCE working electrode, SCE reference electrode and glassy carbon counter electrode.*

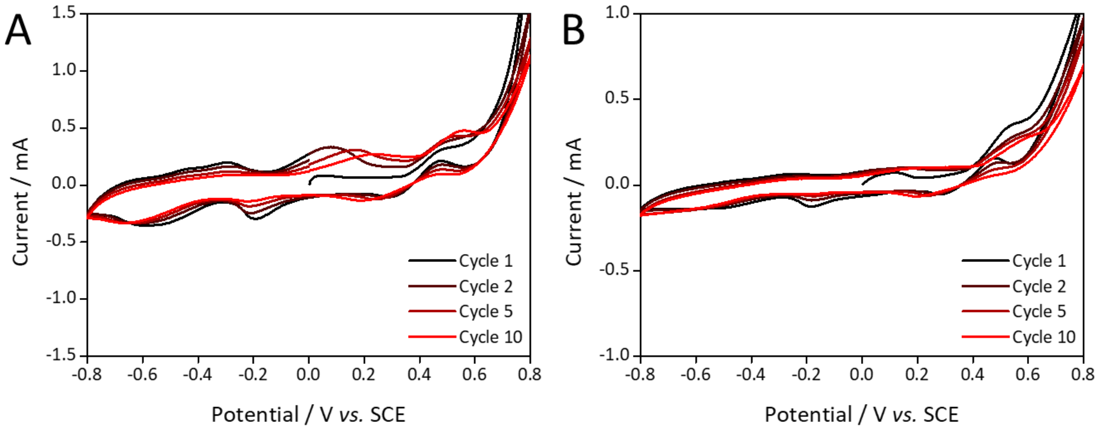

Figure S15. *CVs of (A) **PMo_{12}@SWNT** and (B) **P_2Mo_{18}@SWNT** recorded in 1M NaOH at a scan rate of 0.1 V s^{-1} with a GC working electrode, SCE reference electrode and platinum counter electrode. The near flattening of the redox waves (as compared to Figures S12 and S14, for instance) indicate how measurement in basic solution effectively 'turns off' the redox properties of the system.*

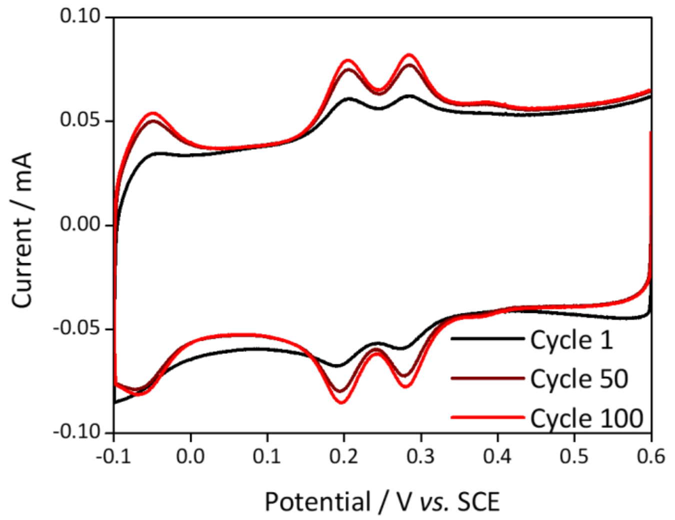

Figure S16. *CVs of* ***PMo_{12}@SWNT*** *recorded in 1M H_2SO_4 after first cycling the POM@SWNT modified electrode for 10 cycles in 1M NaOH. Both cycles (in base and acid) were performed at a scan rate of 0.1 V s^{-1} with a GC working electrode, SCE reference electrode and platinum counter electrode.*

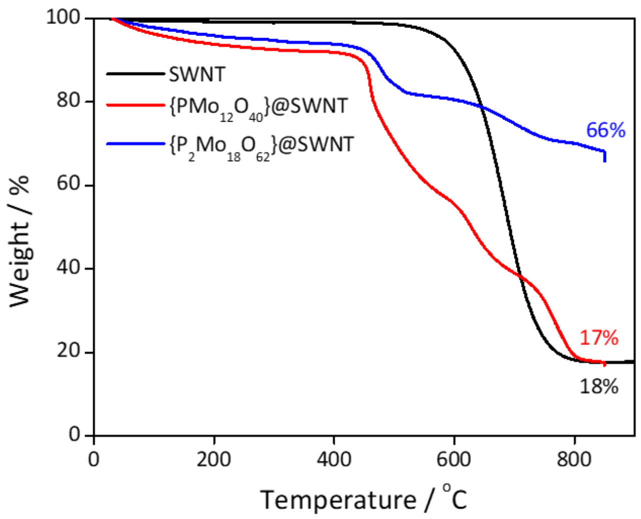

Figure S17. *Thermal gravimetric analysis (TGA) of* ***opened SWNTs, PMo_{12}@SWNT*** *and* ***P_2Mo_{18}@SWNT****. Data acquired under an atmosphere of air (90 mL/min) on platinum pans, at a ramp rate of 10 °C per minute to 1000 °C.*

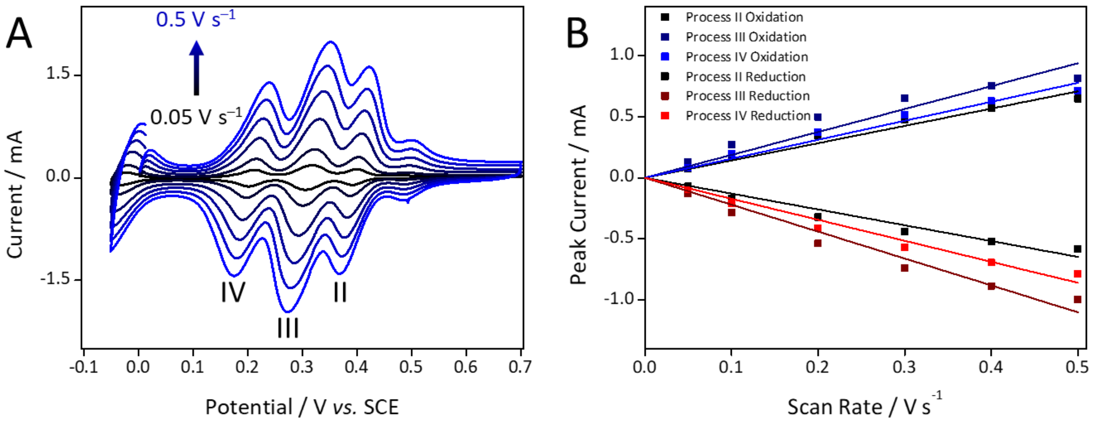

Figure S18. *(A) CVs of* ***P_2Mo_{18}@SWNT*** *recorded at varying scan rates and, (B) Peak current vs. scan rate for the processes shown in A, showing a linear relationship which is indicative of surface confined redox behaviour.*

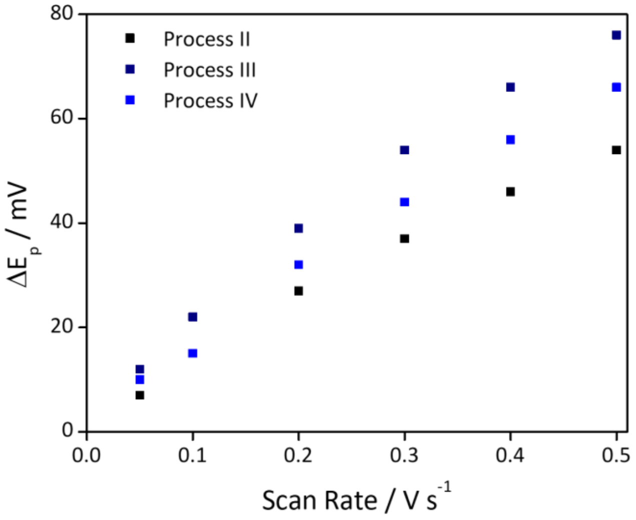

Figure S19. *Peak-to-peak separation (ΔE_p) vs. scan rate for* ***P_2Mo_{18}@SWNT****, confirming the electrochemical quasi-reversibility of the nanotube-confined POM redox processes*

References

[1] A. Linz, *Ind. Eng. Chem. Anal. Ed.* 1943, *15*, 459–459.
[2] L. E. Briand, G. M. Valle, H. J. Thomas, *J. Mater. Chem.* 2002, *12*, 299–304.
